# Wood’s Medical and Surgical Monographs

**Published:** 1891-10

**Authors:** 


Wood's Medical and Surgical Monographs, consisting of original treatises and reproductions in English of books and monographs selected from the latest literature of foreign countries, with illustrations, etc. Published monthly. Vol. XI., No. 1, July; No. 2, August; No. 3, September, 1891. New York: Wm. Wood & Co. $10.00. $1.00 per part.

The July number of the current volume contains the following titles by well-known authors: Hay Fever and Paroxysmal Sneezing, by Sir Morell Mackenzie, M. D.; Tuberculosis of the Bones and Joints, by Dr. Fedor Krause; and, A Study of the Malignant Diseases of the Upper Air Tract, by Francke H. Bosworth, M. D.

The August number contains the following monographs: Modern Abdominal Surgery, by Sir T. Spencer Wells, Bart.; Subjective Noises in the Head and Ears: Their Etiology, Diagnosis and Treatment, by H. MacNaughton Jones, M. D.; Notes on Surgery for Nurses, by Joseph Bell, M. D.; and Surgical Treatment of Typhlitis, by Frederick Treves, F. R. C. S.

The September part brings out the following: Foods and Dietaries: A Manual of Clinical Dietetics, by R. W. Burnett, M. D.; Stertor, Apoplexy, and the Management of the Apoplectic State, by Robert I. Bowles, M. D.; and the Index of Volume XI.

Many of these subjects are copiously illustrated, and all are ably and interestingly handled. This volume bears further testimony to the wisdom and liberality of the publishers.

